# Multi-Ligament Reconstruction in an Adolescent Female Affected by Congenital Femoral Deficiency and Complete Anterior and Posterior Cruciate Ligament agenesis: A Case Report

**DOI:** 10.3390/clinpract15010001

**Published:** 2024-12-24

**Authors:** Simone Giusti, Maria Beatrice Bocchi, Edoardo De Fenu, Osvaldo Palmacci, Ezio Adriani

**Affiliations:** 1 Operative Research Unit of Orthopaedic and Trauma Surgery, Fondazione Policlinico Universitario Campus Bio-Medico, Via Alvaro del Portillo, 00128 Roma, Italy; 2 Complex Operational Unit of Sports Traumatology and Joint Reconstruction, Fondazione Policlinico Universitario Agostino Gemelli IRCCS, Largo Agostino Gemelli 8, 00168 Roma, Italy; edoardo.defenu@policlinicogemelli.it (E.D.F.); ezio.adriani@policlinicogemelli.it (E.A.); 3 Department of Trauma and Orthopaedic Surgery, Università Cattolica del Sacro Cuore, Fondazione Policlinico Universitario Agostino Gemelli IRCCS, Largo Agostino Gemelli 8, 00168 Roma, Italy; mariabeatrice.bocchi01@icatt.it (M.B.B.); osvaldo.palmacci@policlinicogemelli.it (O.P.); 4 Orthopaedics and Traumatology Department, Università Cattolica del Sacro Cuore, Fondazione Policlinico Universitario Agostino Gemelli IRCCS, Largo Agostino Gemelli 8, 00168 Roma, Italy

**Keywords:** congenital femoral deficiency, posterior cruciate ligament, anterior cruciate ligament, agenesis, case report

## Abstract

**Purpose:** Multi-ligament reconstruction in adolescent patients affected by congenital femoral deficiency is an extremely rare and delicate surgical procedure. There are very few reported cases of complete anterior and posterior cruciate ligament agenesis in these patients. **Methods:** We present a complex case of a 16-year-old girl affected by congenital femoral deficiency and ipsilateral tibial hypoplasia who was treated successfully for a complete agenesis of the anterior (ACL) and posterior (PCL) cruciate ligament with single-sitting ACL and PCL reconstruction. **Results:** The adolescent patient was successfully reconstructed with excellent clinical results. **Conclusions:** Knee MRI (Magnetic Resonance Imaging) should be requested in all patients affected by congenital femoral deficiency to exclude ligamentous agenesis. Where present, these should be reconstructed at an early stage as soon as limb-lengthening procedures are completed. If still skeletally immature, physeal-sparing surgical techniques should be implemented.

## 1. Introduction

Congenital femoral deficiency (CFD) is a rare genetic condition with an incidence of 1.1–2.0 in 100,000 live births [1], which encompasses a variety of paediatric developmental deformities of the femur bone [2]. This condition often results in significant leg length discrepancy, deformity, and functional deficits [3]. These patients are often also found to have distal femoral valgus deformities, medial femoral condylar hypoplasia, soft tissue contractures and, most importantly, anteroposterior knee instability due to congenital agenesis of the anterior (ACL) and posterior (PCL) cruciate ligament [4]. Patients affected by CFD are commonly treated with leg-lengthening procedures [5] at an early stage; however, the resulting anteroposterior knee instability is often overlooked and commonly not diagnosed or only treated if the patient was to become symptomatic. This is considered unacceptable with modern advances in imaging techniques (magnetic resonance imaging) [6], especially as the results of the untreated instability could lead to heavy meniscal damage and early osteoarthritis.

## 2. Case Report

**Figure 1:**
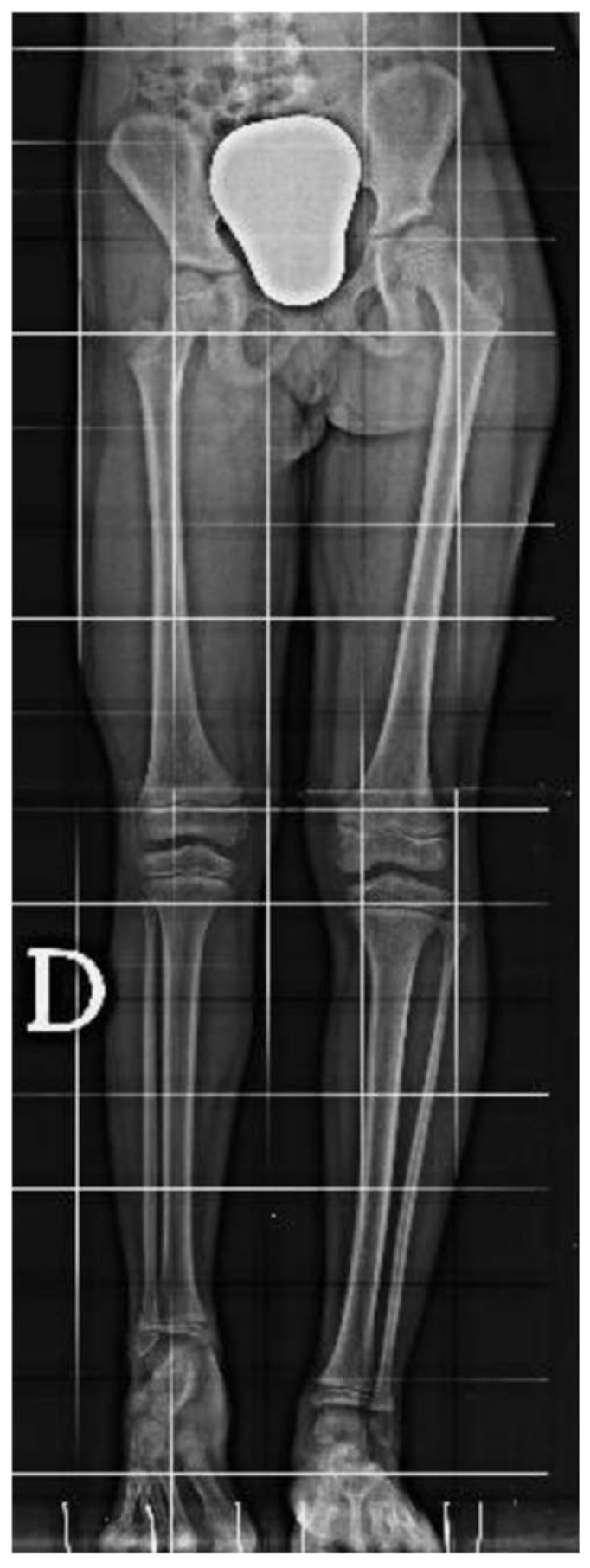
Long-standing plain film radiographs of the patient showing an acquired leg length discrepancy of 75 mm.

An 8-year-old girl presented to a different hospital with a complaint of leg length discrepancy, right shorter than left. She was brought to local medical attention and promptly diagnosed with congenital femoral deficiency and ipsilateral tibial hypoplasia. At this time, the patient had an acquired leg length discrepancy of 75 mm (femoral 50 mm and tibial 25 mm) (Figure 1); she therefore underwent a proximal femoral osteotomy and placement of an LRS^TM^ Orthofix monolateral external fixator on the femur at a different hospital. The procedure was well tolerated, with no complications and adequate surgical wound healing. Seven days post-operatively, she began the lengthening procedure, which consisted of a one-quarter turn four times per day for a total of 1 mm per day. The patient was followed every two weeks for the first month and then monthly with physical examination and plain film radiographs.

**Figure 2:**
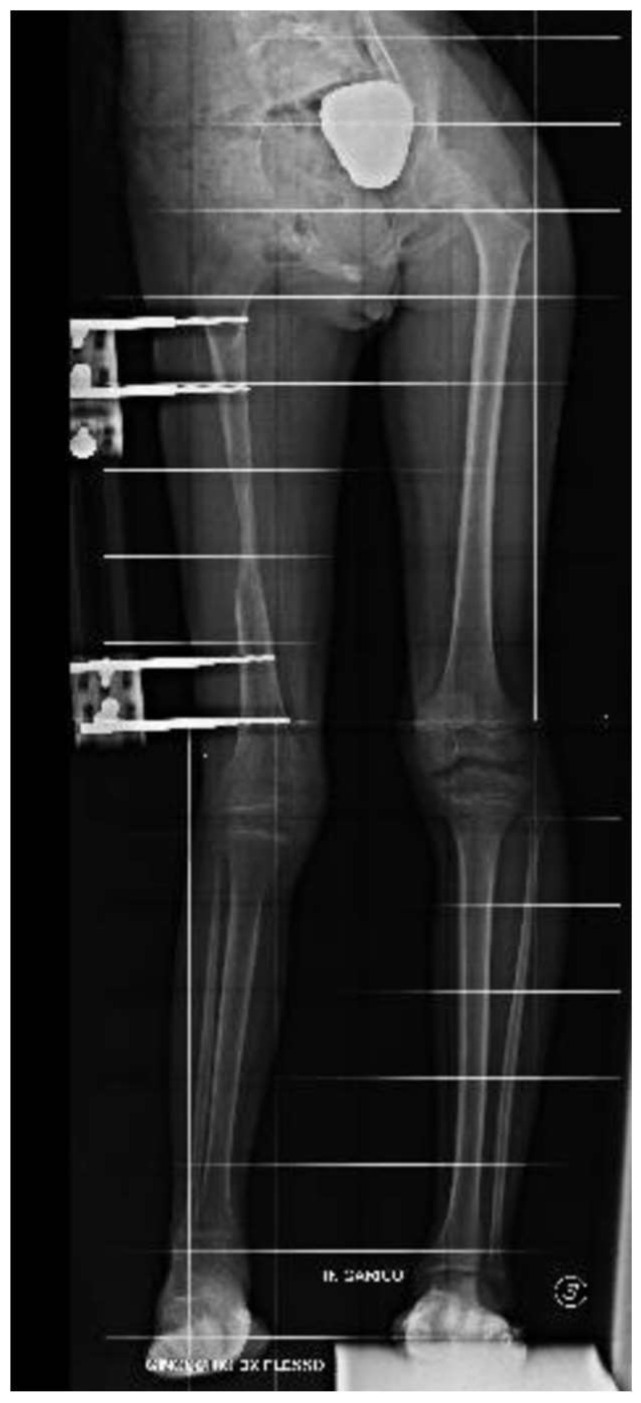
5-month follow up with initial callous formation.

**Figure 3:**
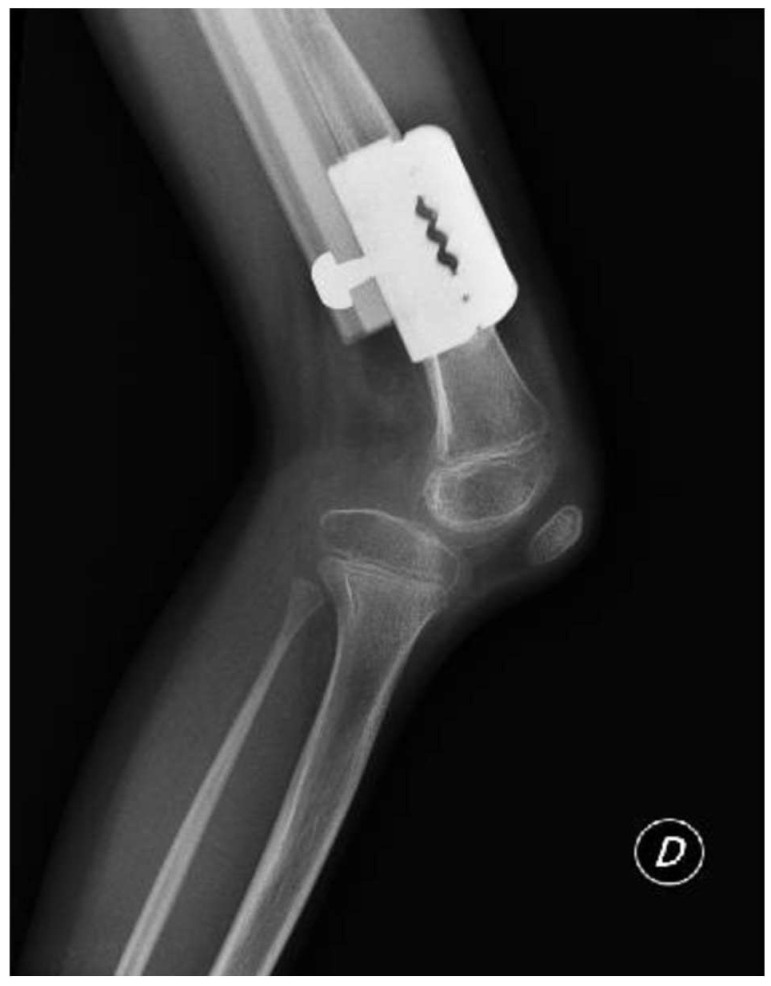
Lateral projection radiographs of the right knee showing posterior subluxation of the tibia with respect to the femoral condyles.

The patient was instructed to start physical therapy for hip and knee range of motion immediately after surgery, while progressive weight bearing was commenced on the last day of lengthening. At 12 weeks, the lengthening goal, 60 mm, was achieved. At 5-month follow up, lower-limb weight-bearing plain radiographs demonstrated that the right femur was within 5 mm of length of the left femur and bone regeneration was visible in the osteotomy gap; however, the study was limited by the inability to fully extend the right knee (Figure 2). Lateral projection radiographs of the right knee showed posterior subluxation of the tibia with respect to the femoral condyles (Figure 3). A potential cause of this posterior subluxation was the 25 mm tibial length discrepancy. No action was taken at the time.

**Figure 4:**
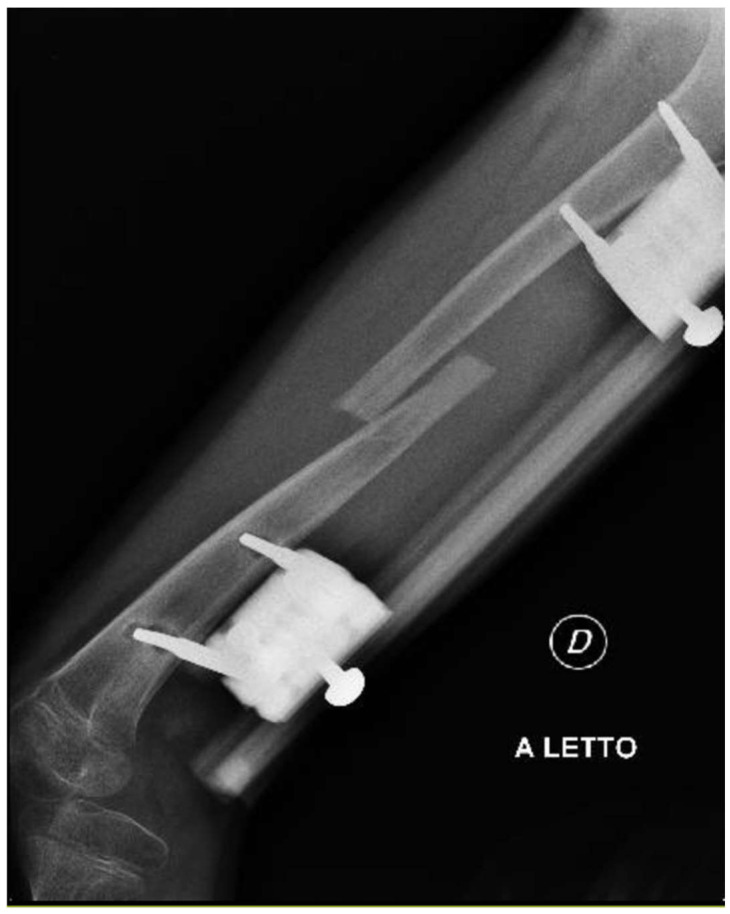
Radiographs showing fracture of the femoral regenerate.

**Figure 5:**
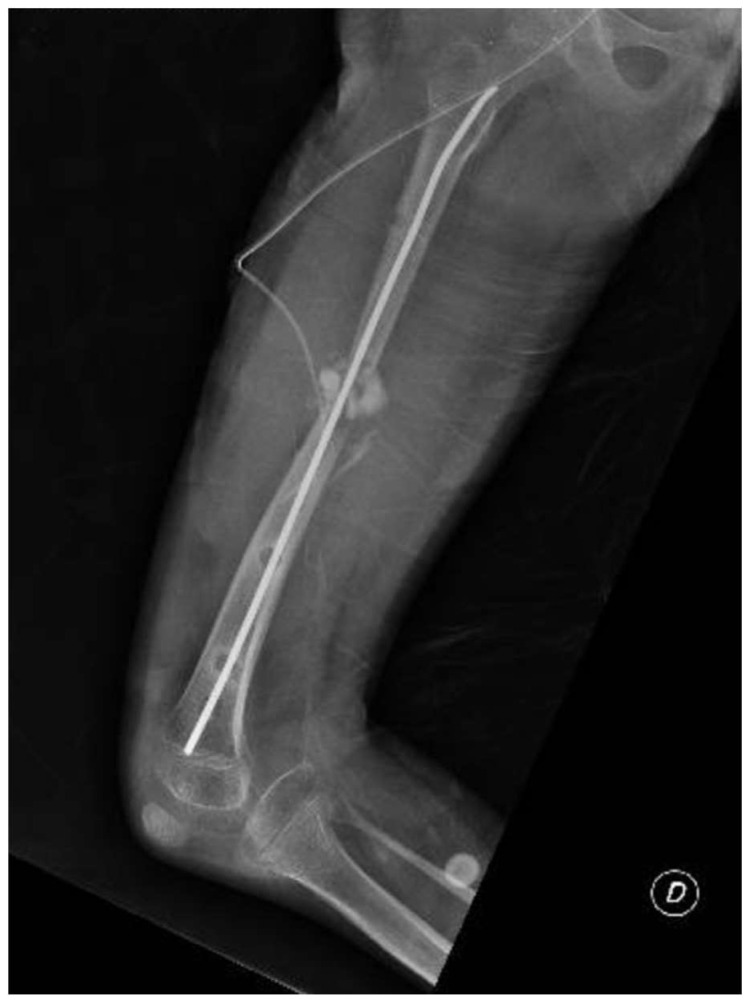
Radiographs showing Elastic Stable Intramedullary Nailing (ESIN) of the femoral fracture.

The patient presented to our attention at about 6 months post-surgery, complaining of acute pain and deformity at the middle third of the thigh. Radiographs showed fracture of the femoral regenerate (Figure 4). The patient underwent immediate external fixator removal, reduction in the knee dislocation, and finally Elastic Stable Intramedullary Nailing (ESIN) of the femoral fracture prior attempt to ream the femoral canal, losing 20 of the 60 mm lengthened (Figure 5). Ten months after the procedure, the patient underwent nail removal for complete fracture consolidation.

**Figure 6:**
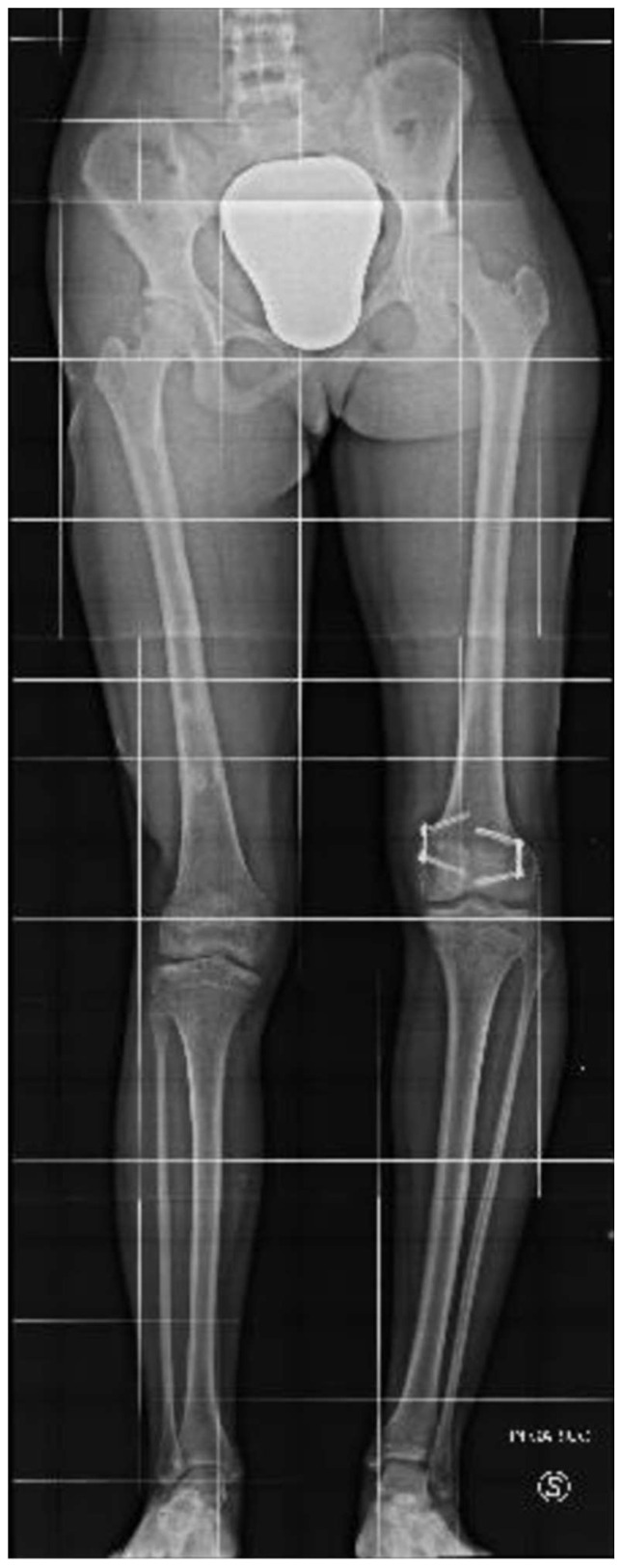
Radiographs showing residual tibial hypoplasia (≅40 mm) and valgus knee deformity.

**Figure 7:**
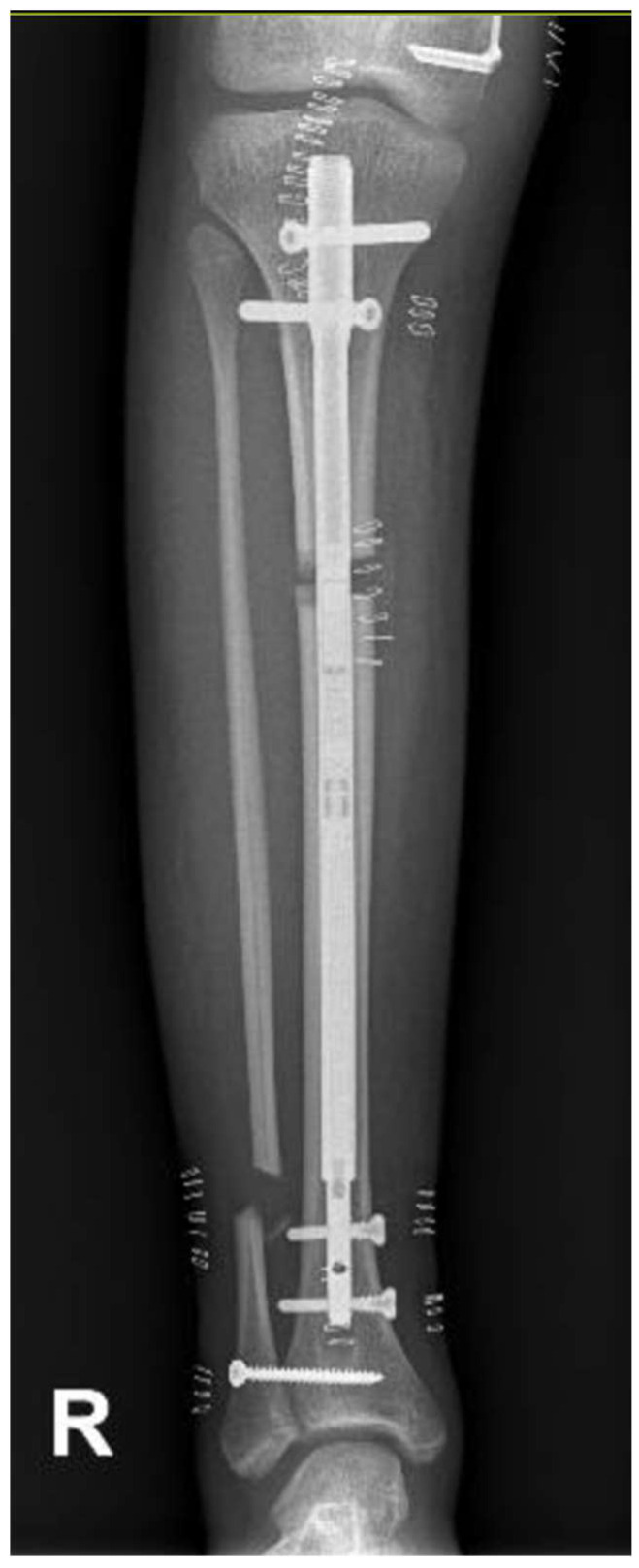
Radiographs showing proximal right tibial osteotomy and placement of an antegrade PRECICE^®^ expandable intramedullary nail and temporary femoral distal medial hemiepiphysiodesis.

**Figure 8:**
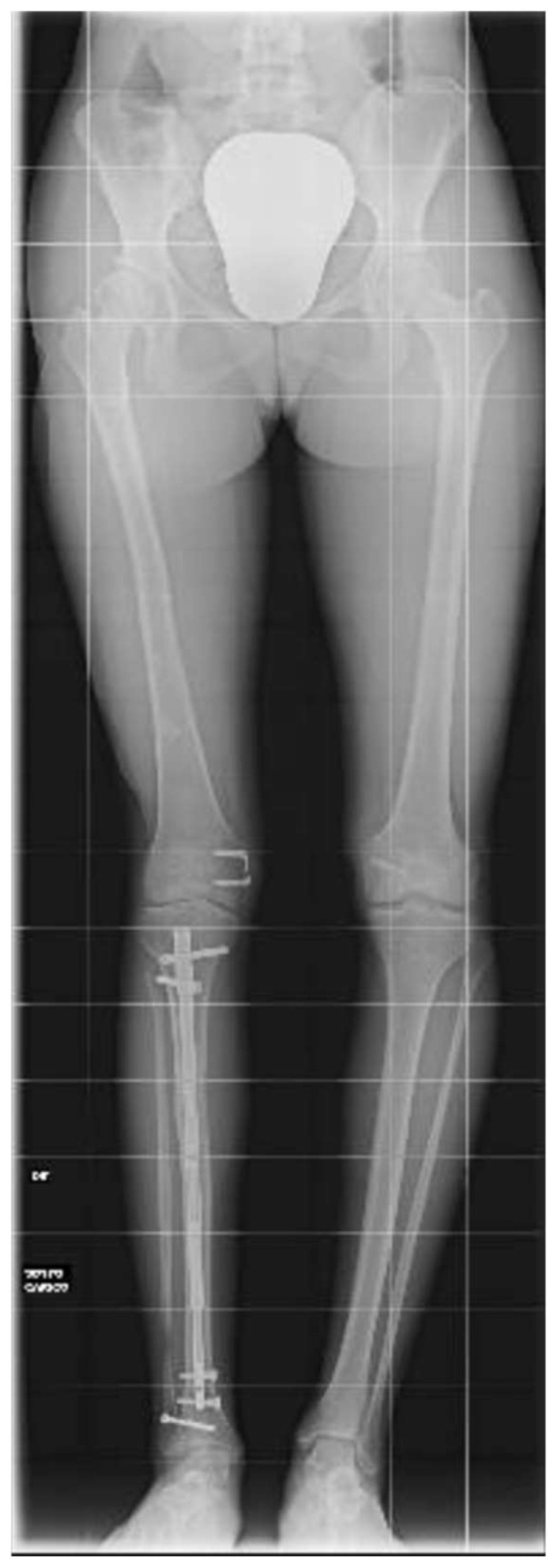
Radiographs showing proximal right tibial osteotomy and placement of an antegrade PRECICE^®^ expandable intramedullary nail and temporary femoral distal medial hemiepiphysiodesis.

At the age of 11, due to the persistent right femoral deficiency, the patient underwent temporary distal femur epiphysiodesis of the left lower limb, thus allowing the length of the two femurs to be evened out in just over 2 years. Finally, at the age of 14, due to residual tibial hypoplasia (≅40 mm) and valgus knee (Figure 6), she underwent a proximal right tibial osteotomy and placement of an antegrade PRECICE^®^ expandable intramedullary nail and temporary femoral distal medial hemiepiphysiodesis (Figure 7 and Figure 8).

**Figure 9:**
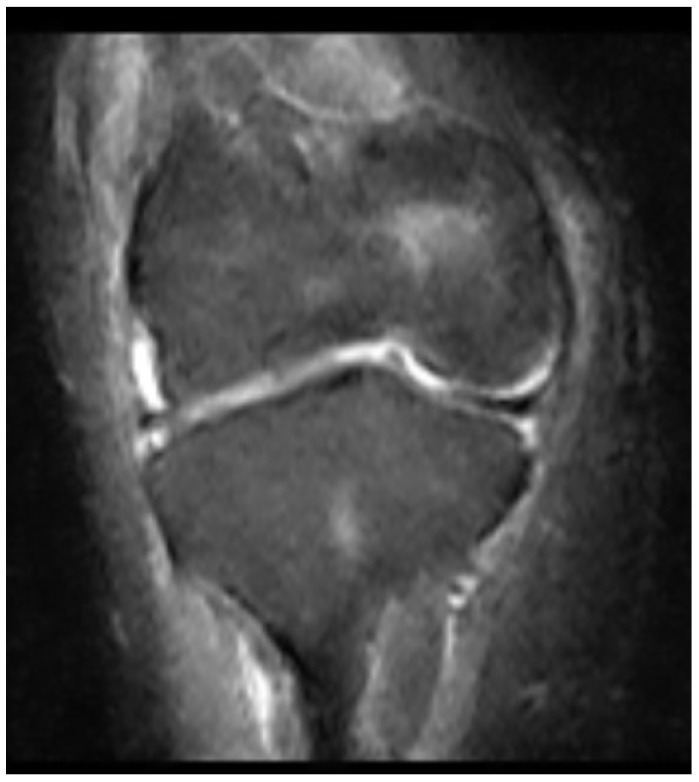
Agenesis of both the anterior and posterior cruciate ligaments.

**Figure 10:**
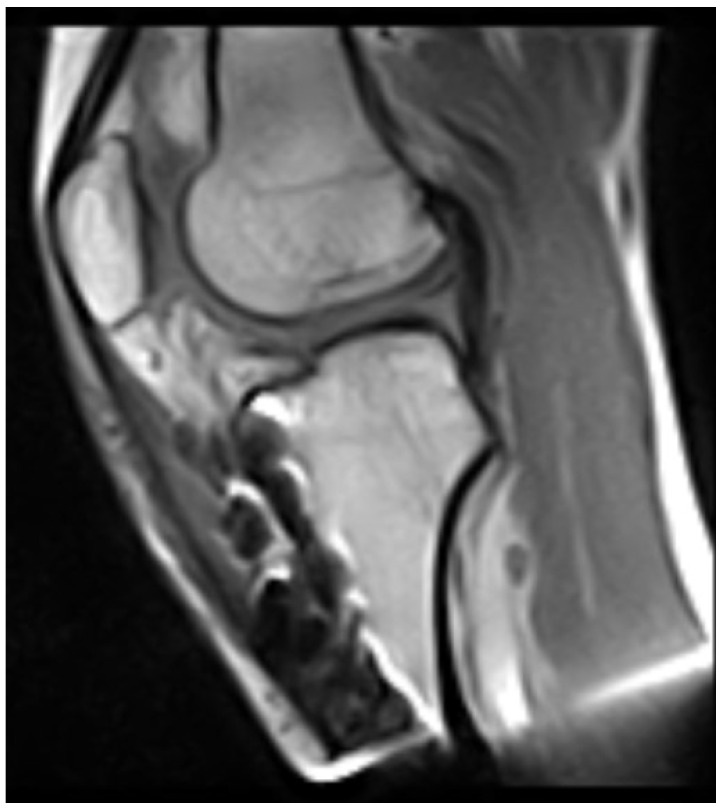
Agenesis of both the anterior and posterior cruciate ligaments.

Then, 2 years later, at the age of 16, the tibial intramedullary nail was removed and, finally, magnetic resonance imaging (MRI) of the knee was acquired. This showed complete agenesis of both the anterior and posterior cruciate ligaments (Figure 9 and Figure 10). In-depth physical examination was carried out on the patient, which revealed severe instability, both anterior (+Lachman and Pivot Shift tests) and posterior (+Posterior drawer test).

After allowing the patient to undergo complete recovery from the limb-lengthening procedure (6 months post-tibial intramedullary nail removal) and carefully evaluating the Tanner scale development of the patient, we decided to reconstruct both cruciate ligaments. The procedure was carried out by an experienced senior surgeon (EA) who reconstructed both ligaments in a single-sitting surgery.

The posterior cruciate ligament was the first to be reconstructed [7].

## 3. Graft Harvesting:

Hamstring tendons were obtained through an incision over the pes anserinus, quadrupled on a graft preparation station, and then secured with high-strength *non-absorbable* suture (*Orthocord* no. 2, Mitek; or Fiberwire no. 5, Arthrex, Naples, FL, USA). The proximal loop was then whip-stitched with vicryl no. 2 to form a tubular graft, and the four ends were prepared with vicryl no. 2. The graft was marked 30 mm from the loop apex using a surgical pen, and its diameter measured.

## 4. Portals

The anteromedial parapatellar portal was established. The anterolateral portal was then formed slightly lower than normal in order to allow the femoral tunnel to be prepared with the in–out technique.

## 5. Joint Preparation

The arthroscope was positioned through the anterolateral portal, while the shaver was introduced through the anteromedial portal to clear any residual tissue from where the ideal femoral attachment site of the PCL should normally be located. The intercondylar fossa was found to be completely closed, with no remnants or malformations of either the ACL or PCL. Meniscal fibrocartilage was present; however, there was total absence of menisco-femoral ligaments, contrary to what is commonly described in similar cases [8].

## 6. Tibial Tunnel Drilling

Using the TransTibial PCL guide and a cannulated reamer sized to match the graft diameter, the tibial tunnel was meticulously drilled. Additionally, a high-strength nonabsorbable passing suture was placed within the tibial tunnel.

## 7. Femoral Half Tunnel Drilling

Identification of where the native PCL footprint would have been on the lateral surface of the medial femoral condyle initiated the femoral half tunnel drilling. The femoral aimer was then positioned through the lower anterolateral portal, and the in–out drilling technique with a cannulated reamer matching the graft diameter was employed. The femoral half tunnel was drilled to a depth of 27 mm to accommodate the curve guide, ensuring precision in the surgical process.

## 8. Fixation Preparation with Curve Cross-Pin System

Inserting the curve guide frame through the lower anterolateral portal, the femoral rod was placed in the femoral half tunnel until the 27 mm marking was reached. Accurate placement of the guide block on the lateral femoral condyle is essential for trocar, sleeve, and pin insertion. This technique positions the guide block 2.5 cm anterior and 2.5 cm proximal to the lateral epicondyle. The arc attachment was assembled, and the bone stock evaluated using a bone gauge pin against the medial femoral condyle cortex. Trocars were inserted from the lateral femoral condyle downward and laterally. A passing suture through the femoral rod size was placed, and the bone gauge pin was used through trocars for visual confirmation of correct positioning within the half tunnel.

## 9. Graft Passage

Tibial and femoral passing sutures were retrieved through the anterolateral portal. A loop was created in the femoral passing suture, with the tibial passing suture threaded through. The loop, housing the tibial passing suture, was then pulled out of the femoral tunnel as a single passing suture. The graft was positioned on the passing suture at the tibial tunnel entrance and smoothly passed through with assistance from a blunt instrument in the posteromedial portal.

**Femoral Fixation:** The graft was secured on the femur using pins, ensuring alignment with the trocar, and verifying the laser line alignment.

**Tibial Fixation:** The knee underwent cycling through flexion and extension to condition the graft. At 70° of knee flexion, the graft was securely fixed using a 2 mm interference screw.

We then proceeded with reconstructing the anterior cruciate ligament.

## 10. Graft Preparation

The graft was prepared for fixation by a suspensory mechanism with an ULTRABUTTON (Smith & Nephew, Andover, MA, USA) at one end.

## 11. Tibial Tunnel Drilling

Using the TransTibial ACL guide and a cannulated reamer sized to match the graft diameter, the tibial tunnel was drilled. Additionally, a high-strength nonabsorbable passing suture was placed within the tibial tunnel.

## 12. Femoral Half Tunnel Drilling

The ACL footprint was identified on the neo-medial surface of the lateral femoral condyle. The femoral anteromedial aimer 5.5 mm was then positioned through the lower anteromedial portal, and with the knee flexed at 110° we passed a Kirschner guide wire. We used a 4.5 mm reamer to create a full tunnel for the femoral suspension fixation; the femoral half tunnel was then drilled to a depth of 23 mm to accommodate the graft with an 8.5 mm reamer.

## 13. Graft Passage

Tibial and femoral passing sutures were retrieved through the anteromedial portal. A loop was created in the femoral passing suture, with the tibial passing suture threaded through. The loop, housing the tibial passing suture, was then pulled out of the femoral tunnel as a single passing suture. The graft was positioned on the passing suture at the tibial tunnel entrance and passed through the tunnels.

## 14. Femoral Fixation

The graft was secured on the femur using the suspension fixation with ULTRABUTTON (Smith & Nephew, Andover, MA, USA).

## 15. Tibial Fixation

The knee underwent cycling through flexion and extension to condition the graft. At 30° of knee flexion, the graft was securely fixed using a 10 × 25 mm interference screw BIOSURE REGENESORB (Smith & Nephew, Andover, MA, USA).

A surgical drain was inserted through the AL portal and secured. Vicryl rapid sutures were used for skin closure. No intra-operative complications were reported, with a total surgical time of 63 min from incision to final suture. The total tourniquet time was 45 min.

The patient underwent physical rehabilitation according to standard protocols and made a full recovery. Clinical follow-up showed complete knee range of motion and negative anterior and posterior drawer tests. Magnetic resonance imaging (MRI) at 6 months post-operation showed correct graft positioning and maturation.

## 16. Discussion and Conclusions

ACL and PCL reconstruction in paediatric patients remains a highly controversial topic due to the supposed risks of damaging the physis when forming the tibial and femoral tunnels [9]. However, the recent literature has shown how early surgical intervention is to be preferred to delayed treatment, as this carries more risks of knee instability, delayed return to sports and activities of daily living and, most importantly, meniscal damage and early cartilage wear [10]. These principles should also be applied to young patients affected by congenital femoral deficiency. In the case we reported, the patient was a 16-year-old with Tanner stage IV; therefore, there was no doubt about the necessity of immediate multi-ligament reconstruction.

Single-stage ACL and PCL reconstruction versus two-stage is debatable and much related to surgeon preference and experience; however, where possible, single-stage should be preferred to avoid unnecessary anaesthesia on a paediatric patient and in order to allow for a prompt return to normal activities following a single period of intensive rehabilitation.

In very young patients with open physis, the reconstruction should be carried out via physeal-sparing techniques such as over-the-top [11] or all-inside [12] surgical techniques to reduce the risk of physeal damage.

For patients affected by congenital femur deficiency, whether this has already been diagnosed or is suspected, a knee MRI (1.5 Tesla or superior) should always be requested to exclude ligamentous agenesis and to avoid knee subluxation during limb-lengthening procedures; this is also a safe diagnostic technique as it carries no added radiation risk for the young patient. Alternatively, where requesting an MRI is not feasible, a full knee exam to exclude anterior–posterior knee instability (Anterior drawer test, Lachman test, and Jerk test) should be performed in all paediatric patient candidates for limb lengthening—these should always be carried out comparatively with the other limb and caution should always be taken, as paediatric patients can often have a congenital knee laxity, which could be mistaken for an incompetent cruciate ligament. Limb-lengthening procedures should always take precedence over ligamentous reconstruction, which should be carried out once complete and acceptable skeletal maturity (minimum Tanner 4) is reached.

When carrying out the limb-lengthening procedures, extreme caution should be taken to avoid dislocating the knee joint. In severe cases of joint instability, the application of a hinged-knee external fixator could be considered with caution.

This case report provides an insightful view on how rare cases like the one described should be treated, confirming that simultaneous reconstruction of both cruciate ligaments is not only possible in these scenarios, but absolutely fundamental in order to stabilise the knee joint and, as a result, avoid long-term complications such as meniscal and cartilage damage.

## Data Availability

All data can be made available upon reasonable request.
